# Regulation of the Golgi Apparatus by p38 and JNK Kinases during Cellular Stress Responses

**DOI:** 10.3390/ijms22179595

**Published:** 2021-09-04

**Authors:** Cathrine Nordgaard, Maxim A. X. Tollenaere, Ana Martinez Del Val, Dorte B. Bekker-Jensen, Melanie Blasius, Jesper V. Olsen, Simon Bekker-Jensen

**Affiliations:** 1 Center for Healthy Aging, Department of Cellular and Molecular Medicine, University of Copenhagen, Blegdamsvej 3B, 2200 Copenhagen, Denmark; nordgaard@sund.ku.dk (C.N.); EVXDK@leo-pharma.com (M.A.X.T.); blasius@sund.ku.dk (M.B.); 2 LEO Pharma A/S, Industriparken 55, 2750 Ballerup, Denmark; 3 Mass Spectrometry for Quantitative Proteomics, Proteomics Program, The Novo Nordisk Foundation Center for Protein Research, Faculty of Health and Medical Sciences, University of Copenhagen, Blegdamsvej 3B, 2200 Copenhagen, Denmark; ana.mdval@cpr.ku.dk (A.M.D.V.); dorte.bekker-jensen@cpr.ku.dk (D.B.B.-J.); jesper.olsen@cpr.ku.dk (J.V.O.)

**Keywords:** stress signaling, phosphorylation, p38, JNK, GIGYF, translation and Golgi

## Abstract

p38 and c-Jun N-terninal kinase (JNK) are activated in response to acute stress and inflammatory signals. Through modification of a plethora of substrates, these kinases profoundly re-shape cellular physiology for the optimal response to a harmful environment and/or an inflammatory state. Here, we utilized phospho-proteomics to identify several hundred substrates for both kinases. Our results indicate that the scale of signaling from p38 and JNK are of a similar magnitude. Among the many new targets, we highlight the regulation of the transcriptional regulators grb10-interacting GYF protein 1 and 2 (GIGYF1/2) by p38-dependent MAP kinase-activated protein kinase 2 (MK2) phosphorylation and 14–3–3 binding. We also show that the Golgi apparatus contains numerous substrates, and is a major target for regulation by p38 and JNK. When activated, these kinases mediate structural rearrangement of the Golgi apparatus, which positively affects protein flux through the secretory system. Our work expands on our knowledge about p38 and JNK signaling with important biological ramifications.

## 1. Introduction

Inflammation is associated with systemic responses to infection, toxins and environmental stresses. The five pillars of systemic inflammation are redness, swelling, pain, heat and local loss of bodily functions. These alterations are largely caused by the action of local as well as systemically acting cytokines which are produced and secreted by cells of the immune system [1]. The mitogen-actived protein (MAP) kinases p38 and c-Jun N-terninal kinase (JNK) are central transducers of inflammatory and cellular stress ignaling pathways. There are three isoforms of the p38 family and three members of the JNK family, all of which are encoded by distinct genes and with tissue-specific expression patterns [2]. These kinases are activated both by cytokines and a number of stress agents, such as ribosomal dysfunction, UV light, oxidative stress and heat or osmotic shock [3]. MAP kinases are generally activated through signal transduction cascades involving upstream MAP kinase kinases (MAPKKs/MKKs) and MAP kinase kinase kinases (MAPKKKs). For the p38 axis, much of the downstream signaling is channeled through p38-activated kinases, such as MAP kinase-activated protein kinase 2/3 (MK2/3), MAPK-interacting kinases 1/2 (MNK1/2) and mitogen and stress activated protein kinase 1/2 (MSK1/2) [4]. Besides mediating acute responses to infection, low-grade inflammation is a hallmark of cancer, aging and a range of human pathologies [5]. A key role for MAP kinases in inflammatory signaling is to mediate the production of new cytokines that, when secreted from cells, will amplify cellular communication and immune functions. Specifically, it is well established that p38 and its downstream kinase MK2 positively regulate the transcription of cytokine genes as well as the stabilization and translation of the derived mRNAs [4].

In light of the plethora of agents and cellular perturbations that trigger p38 and JNK activation, it is not surprising that these kinases play diverse roles in re-shaping cellular function. This is especially evident upon stress stimuli that cause strong and prolonged MAP kinase activation. Next to mounting an appropriate inflammatory response, p38 and JNK signaling is in most cells inhibitory to cell cycle progression and positively regulates cell death pathways [3]. In other organismal and cellular contexts, these kinases mediate differentiation and regulate metabolic pathways [6]. MAP kinases exert their dominance on cellular functions through the phosphorylation of numerous substrates that collectively reshape cellular physiology. Such regulation allows cells to cope with acute stress and/or mounting inflammatory responses. Some of these modifications have well-understood consequences, such as the p38-regulated depletion of centriolar satellites [7,8]. The consequences of the majority of the modification events, however, are more elusive. Many of them may simply constitute subtle re-shaping of biochemical activities through the modification of multiple pathway components. This mode of action is similar to how cyclin-dependent kinase (CDK) activity reshapes the signaling landscape of mitotic cells [9], and how ataxia telangiectasia mutated serine/threonine kinase (ATM) exerts its dominance over multiple cellular functions during the DNA damage response [10]. Despite the recognized importance of p38- and JNK-signaling for cellular physiology, only few focused attempts to identify the full inventory of substrates of these kinases have been made. With a few but notable exceptions, our knowledge about specific substrates comes largely from targeted studies of specific phosphorylation events. To date, attempts to grasp the full breath of MAP kinase substrates have only been successful for p38 and its downstream kinase MK2 [11]. As a consequence, our knowledge about the full scope of JNK signaling is lacking behind [12]. In the present work, we set out to remedy this discrepancy and map new phosphorylation reactions mediated by this kinase. Our results indicate that the number of cellular substrates of JNK kinases is similar in abundance to those of p38 and its downstream kinases.

Protein secretion is achieved through highly coordinated modification and sorting of proteins through the endoplasmic reticulum (ER) and Golgi apparatus. Secreted proteins are packaged in vesicles that bud off from the ER and fuse with membranes of the *cis* face of the Golgi network [13]. Proteins travel through the cisternae of the *cis*, *medial* and *trans* Golgi network. Finally, secretory vesicles released from the *trans* Golgi network fuse with the cell membrane to release their content to the cell exterior [14]. On their journey through the secretory system, cargo proteins are extensively modified in complex glycosylation reactions, and these modifications may impact on both stability and function of secreted proteins [15]. The Golgi apparatus is famous for its remarkable structural plasticity and dynamic properties. Most notably, the biochemical environment of mitotic cells enforce a complete fragmentation of the structure, which is key to both cell division itself and partitioning of this essential organelle to the daughter cells [16].

The secretory system is of utmost importance for cell-to-cell communication by the immune system, and its capacity can be enhanced in specialized cells and during immune cell maturation. For example, antibody-producing plasma cells contain excessive Golgi networks to allow for their abnormally high flux of protein secretion [17]. Similarly, inflammatory stimulation of cytokine producing cells may be associated with the enhancement of secretion capacity. However, our knowledge of the molecular details governing regulation of Golgi structure and function during inflammation is lacking behind our knowledge of the upstream signaling events and the mechanisms and consequences of cytokine production. Here, we show that inflammatory signals and cellular stress insults regulate both the structure and function of the Golgi apparatus in a manner dependent on both p38 and JNK activity. This regulation appears to be achieved through modification of multiple Golgi-resident proteins and allows for enhanced protein flux through the secretory system.

## 2. Results

**Figure 1:**
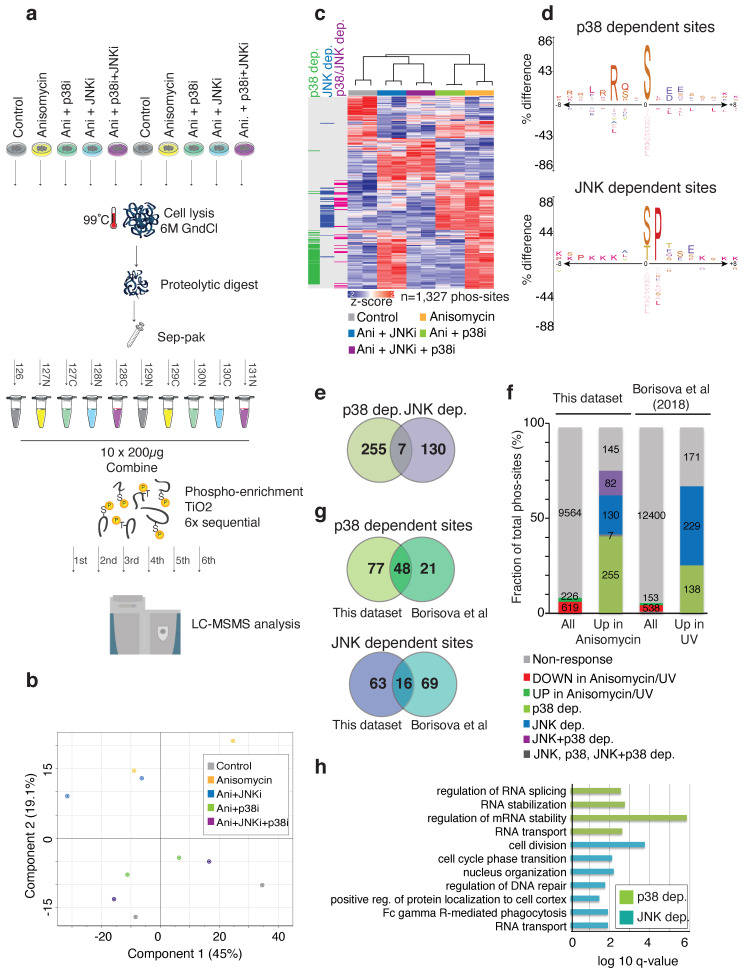
Phospho-proteomic analysis of p38- and JNK-dependent phosphorylation reactions. (**a**). Schematic of phospho-proteomic workflow. U2OS cells were treated in duplicates with indicated combinations of anisomycin (Ani, 1 h) and p38 and JNK inhibitors (p38i, JNKi, 0.5 h pre-treatment). Lysates were digested with trypsin and peptides were labeled with 10-plex Tandem Mass Tags (TMT). Phospho-peptides were enriched and analyzed by mass spectrometry. (**b**). Principal component analysis on datasets obtained from (**a**). (**c**). Heatmap with clustering of phospho-sites differentially regulated by anisomycin treatment (absolute fold change >0.5, FDR 5%). Intensity is represented as z-score. Anisomycin-increased sites that were dependent on JNK kinase activity (blue) and p38 kinase activity (green) or exclusively downregulated in samples treated with both inhibitors (purple) are highlighted (absolute fold change >0.5, FDR 5%). (**d**). Sequence motifs of p38- and JNK-dependent phosphorylation sites from (**c**). (**e**). Venn diagram showing the overlap between p38- and JNK-dependent phospho-sites from (**a**,**c**). (**f**). Numbers of mapped phosphorylation sites from (**a**) compared to a published dataset with UV irradiation and p38 inhibition [11]. Numbers of anisomycin- or UV-induced phosphorylation sites are broken down into groups based on inhibitor effects. For the UV-dataset, UV-upregulated and p38-independent sites with proline in the +1 position were scored as “JNK-dependent”. ‘JNK+p38 dep.’ are phosphorylation sites that are only significantly downregulated in the ‘Ani+p38 i+JNKi’ condition compared to the anisomycin condition. ‘JNK, p38 and JNK+p38 dep.’ are phosphorylation sites that are significantly downregulated in all three inhibitor treated conditions. (**g**). Top: Venn diagram showing the overlap between the common p38-dependent sites from our data and [11]. Bottom: Venn diagram showing the overlap between the common JNK-dependent sites from our data and UV-upregulated p38-independent pS/pTP sites from [11]. (**h**). GO and KEGG terms enriched among proteins with p38- (green) and JNK- (blue) dependent phosphorylation sites.

In an effort to identify additional p38- and JNK-regulated phosphorylation targets, we performed TMT (Tandem Mass Tag) peptide-labelling and phospho-proteomic screening in U2OS cells treated with anisomycin and inhibitors of p38 or JNK (Figure 1a). More than 10,000 phospho-sites were identified and quantified in all samples. Principal component analysis revealed a clear separation between conditions (Figure 1b). A total of 619 phospho-sites were found to be differentially up-regulated in response to anisomycin. The analysis revealed that p38- or JNK inhibitors restored initial values of most of these anisomycin-regulated phosphorylation sites (474 out of 619 sites), suggesting that these two kinases are responsible for the large majority of anisomycin-induced phosphorylation changes (Figure 1c; Table S1). We identified substrate-kinase dependency by evaluating which anisomycin-upregulated phosphorylation sites were downregulated upon p38 inhibition (green), JNK inhibition (blue) and combined inhibitor treatment (purple) (Figure 1c; Table S1). However, the majority of the sites in the latter category (purple) clustered as either p38- or JNK-dependent (59 out of 82 sites), suggesting that they are only regulated by one of the two kinases (Figure 1c and Figure S1a). The large majority of p38-dependent phospho-sites contained arginine in the -3 position, which conforms to the consensus for p38-dependent kinases such as MK2. (Figure 1d). The remaining p38- and JNK-dependent phospho-sites were embedded within the proline-directed phosphorylation motifs expected for MAP kinases (Figure 1d; Figure S1b). A breakdown of the absolute numbers of phosphorylation sites allowed us to classify a total of 255 p38-dependent sites and 130 JNK-dependent sites from our phospho-proteomic investigation, for which there was very little overlap (Figure 1e,f). We proceeded to compare our results with those obtained in a published screen [11], where UV-irradiation was used as the p38 and JNK activating stimulus and only p38-dependent sites were characterized. Based on the analysis of our own screen (Figure 1c,d), and the fact that no other proline-directed kinases are known to be activated by UV-irradiation, we surmised that UV-upregulated, p38-independent and proline-directed phosphorylation sites from [11] could also be JNK targets (Figure 1f). While the total amount of p38- and JNK-directed phosphorylation sites were similar between the two datasets, only a smaller subset of targets were found in both screens (Figure 1g). GO and KEGG term enrichment highlighted that p38-dependent substrates were associated with RNA-regulating processes, while JNK-dependent substrates were involved in cell cycle regulation and other processes (Figure 1h). In conclusion, our phospho-proteomic investigation considerably expands the known inventory of JNK targets and highlights the pervasive cellular roles of p38 and JNK upon cellular stress insults.

### 2.1. GIGYF1 Is Phosphorylated and Bound by 14–3–3 after Stress

**Figure 2:**
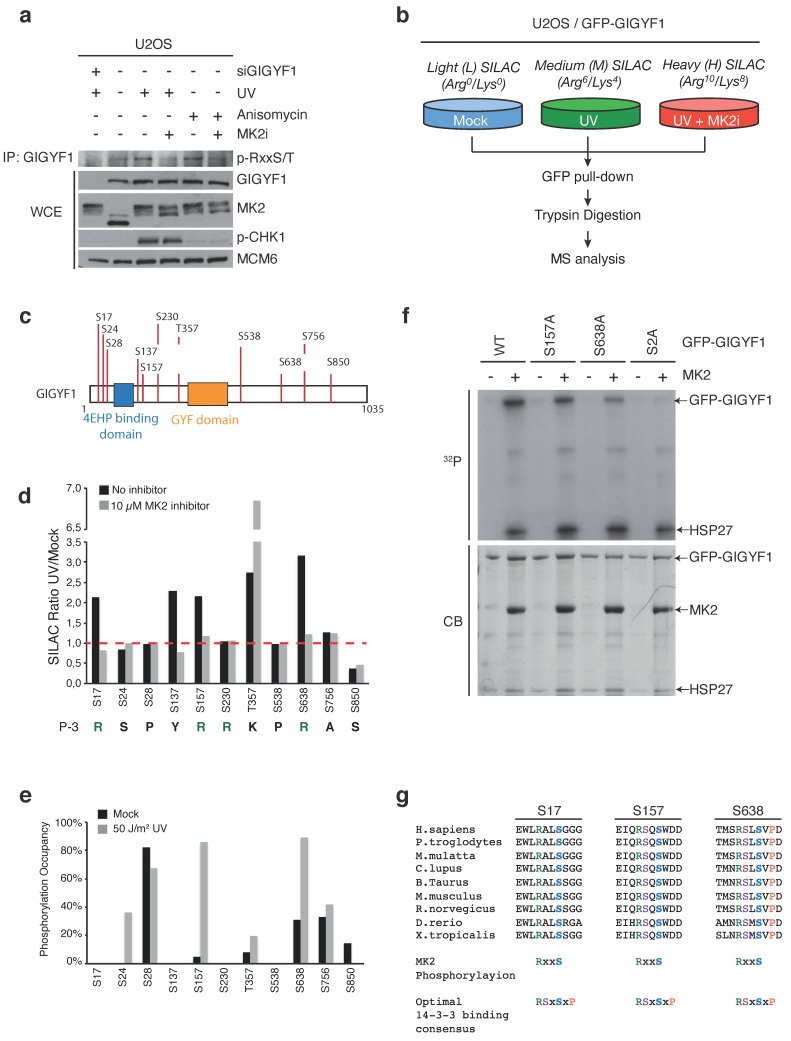
MK2 phosphorylation and 14–3–3 binding of GIGYF1. (**a**). U2OS cells transfected with GIGYF1 siRNA were pre-treated with MK2 inhibitor (MK2i, 0.5 h) and treated with UV (50 J/m^2^, 1 h recovery) or anisomycin (1 h) as indicated. GIGYF1 immuno-precipitated material (IP: GIGYF1) and whole cell extracts (WCE) were analyzed by immunoblotting with the indicated antibodies. (**b**). Schematic of SILAC-based proteomics experiment. U2OS cells stably expressing GFP-GIGYF1 were pre-treated with MK2 inhibitor (0.5 h) and UV-irradiated (50 J/m^2^, 1 h recovery) as indicated. GFP pulldown material was digested with trypsin and subjected to mass spectrometric (MS) analysis. (**c**). GIGYF1 phosphorylation sites identified from (**b**). (**d**). SILAC ratios of GIGYF1 phosphorylation sites from (**b**), calculated as fold change to control (light label). The identity of the amino acid in position −3 is indicated below. (**e**). Occupancy of GIGYF1 phosphorylation sites in mock and UV-treated samples from (**b**). (**f**). Wildtype or alanine-substituted versions of GFP-GIGYF1 were purified from U2OS cells, mixed with recombinant HSP27 and used as substrates for phosphorylation by recombinant MK2. Reactions were resolved by SDS-PAGE and analyzed by autoradiography (^32^P) and coomassie blue (CB) staining. (**g**). Alignment of GIGYF1 phosphorylation sites from multiple species. Optimal consensus sequences for MK2 phosphorylation and 14–3–3 binding is indicated. H., homo; P., pan; M., macaca; C., canis; B., bos; M., mus; R., rattus; D., danio; X., xenopus.

Among the many proteins for which we mapped p38- and/or JNK-regulated phosphorylation sites (Table S1), we took notice of grb10-interacting GYF protein 1 and 2 (GIGYF1 and GIGYF2). Data mining of our own as well as published screens with chemical inhibitors of p38 and MK2 [11] suggested to us that GIGYFs are targeted for phosphorylation by MK2 upon cell stress stimuli with a potential for regulation of the above processes. We managed to corroborate this by probing immunoprecipitated endogenous GIGYF1 with a phospho-specific antibody raised against the MK2 consensus sequence (Figure 2a). Next, we constructed a U2OS cell line with doxycycline-inducible expression of GFP-tagged GIGYF1. Using Stable Isotope Labeling by Amino acids in Cell culture (SILAC), GFP pull-down and mass spectrometry, we analyzed the UV light-induced and MK2-dependent phosphorylation sites on GIGYF1 (Figure 2b–d). A total of four phosphorylation sites were increased after UV in an MK2-dependent manner; S17, S137, S157 and S638 (Figure 2d). Most of these sites conformed to the known MK2 consensus sequence RxxS/T (Figure 2d) and two of them, S157 and S638, displayed a close to 100% phosphorylation site occupancy (Figure 2e). To confirm that MK2 was indeed the responsible kinase, we adopted an in vitro kinase assay approach. To this end, we purified four overlapping and GFP-tagged fragments of GIGYF1 from transfected U2OS cells (Figure S2a) and subjected them to phosphorylation by recombinant MK2. Only fragment 1 and fragment 3 containing S17, S137, S157 and S638, respectively, were phosphorylated in this assay (Figure S2b). Mutation of these two serines completely abolished phosphorylation of the relevant two fragments without interfering with heat shock protein 27 (HSP27) phosphorylation in the same reactions (Figure S2c). In the full-length context, substitution of S157 and S638 to alanines had little effect on their own, but in combination nearly abolished MK2 phosphorylation (Figure 2f). The amino acid sequences surrounding these two phosphorylation sites were conserved through mammalian and vertebrate evolution, whereas the flanking distal regions were less conserved (Figure 2g and Figure S2d). While they are also clearly present in GIGYF2 (Figure S2e), the much lower MK2 phosphorylation efficiency in vitro precluded us from performing a similar detailed analysis for this protein (Figure S2f). We conclude that GIGYF proteins are directly phosphorylated by MK2 on two conserved serines upon cellular stress insults.

### 2.2. MK2 Phosphorylation and 14–3–3 Binding Excludes GIGYF1 from p-Bodies

**Figure 3:**
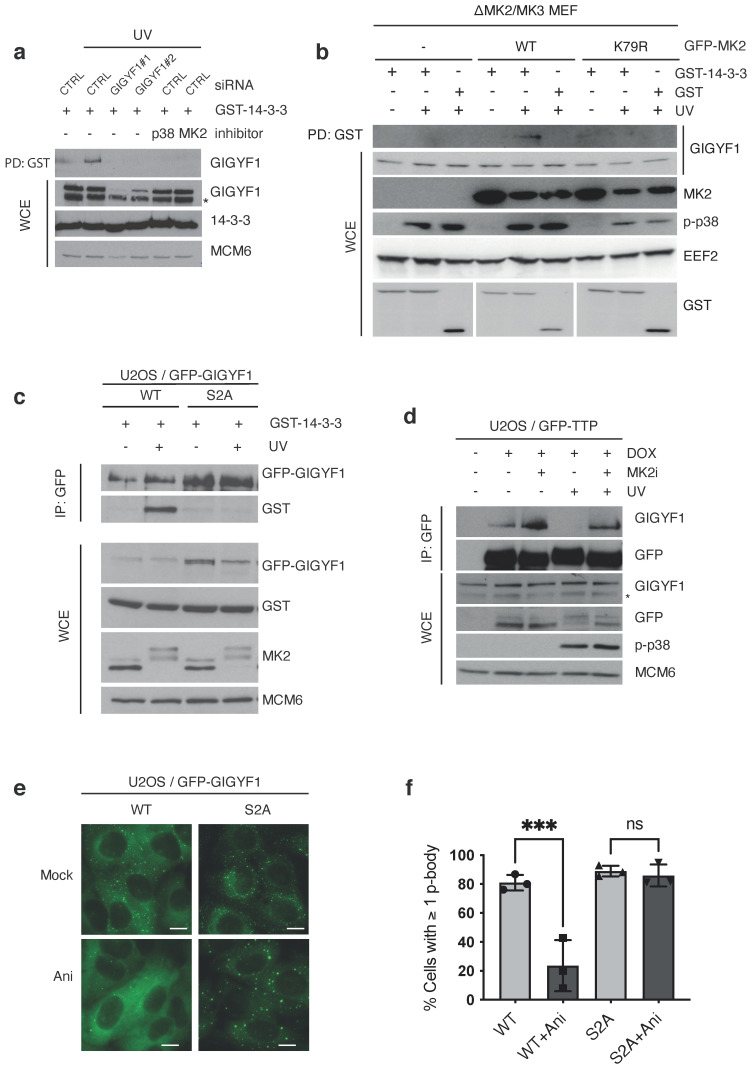
GIGYF1 phospho-mutant retains *p*-body localization upon cellular stress. (**a**). U2OS cells were transfected with the indicated siRNA, pre-treated with p38 and MK2 inhibitors (0.5 h) and UV-irradiated (50 J/m^2^, 1 h recovery) as indicated. Lysates were incubated with recombinant GST-14–3–3 protein or GST alone. GST pull-down material (PD: GST) and whole cell extracts (WCE) were analyzed by immunoblotting with the indicated antibodies. (**b**). Mouse embryonic fibroblasts (MEF) from MK2/MK3 knock-out mice were stably transfected with empty vector (−), wildtype (WT) or kinase-dead (K79R) versions of GFP-MK2. Cells were treated and analyzed as in (**a**). (**c**). U2OS cells stably transfected with wildtype (WT) or phosphosite-mutated (S157A/A638A–“S2A”) GFP-GIGYF were treated as in (**a**). Lysates were incubated with recombinant GST-14–3–3 protein and GFP pull-down material (IP: GFP) and whole cell extracts (WCE) were analyzed by immunoblotting with the indicated antibodies. (**d**). U2OS cells conditionally expressing GFP-TTP were induced by doxycycline (DOX), pre-treated with MK2 inhibitor (0.5 h) and UV-irradiated (50 J/m^2^, 1 h recovery). GFP pull-down material and whole cell extracts (WCE) were analyzed by immunoblotting with the indicated antibodies. Asterisks denotes an unspecific band. (**e**). Cells from (**c**) were treated with anisomycin (1 h) as indicated. Images of fixed samples were acquired by fluorescence microscopy. Scale bars, 10 μm. (**f**). Quantification of (**f**), showing percentages of cells containing ≥1 p-body +/− SD. *n* = 3 of minimum 30 cells, ns, no significance; ***, *p* < 0.001 using a two-way ANOVA with Tukey’s multiple comparisons test.

We noticed that phosphorylated S638 matches the consensus for 14–3–3 binding with an arginine in position -3 and a proline in position + 2 [18] (Figure 2g). We and others have previously shown that a substantial subset of MK2 phosphorylation events provide a binding platform for 14–3–3 proteins [8,11]. Indeed, upon MK2-activating anisomycin or cycloheximide treatment of U2OS and HEK293 cells, we readily co-purified GIGYF1 with recombinant GST-tagged 14–3–3 proteins (Figure S2g,h). The interaction was also induced by UV radiation in a manner dependent on MK2 and its upstream kinase p38 (Figure 3a,b), and mutation of S157 and S638 to alanines (“S2A” mutant) completely abrogated stress-induced GIGYF1/14–3–3 complex formation (Figure 3c). 14–3–3 binding sites often occur in tandem on proteins, providing affinity for both of the phospho-binding interphases of the 14–3–3 dimer [19]. In the present case, the amino acid sequences flanked by S157 and S638 includes the GYF domain of GIGYF1 (Figure S2e). This raised to us the possibility that 14–3–3 binding could obstinate or modulate the protein-binding properties of this domain. The known interacting partners of this specific GYF domain is the poly-proline stretches in the post-transcriptional regulatory proteins tristetraprolin (TTP) and zinc-finger protein (ZNF598) [20,21]. Indeed, GIGYF1 markedly lost its affinity for these two proteins upon UV-irradiation of cells, in a manner dependent on MK2 kinase activity (Figure 3d and Figure S3a). GIGYF1 also binds the translational repressor eIF4E-homologous protein (4EHP) through an unrelated N-terminal domain (Figure 2c), and this interaction was not perturbed under the same experimental conditions (Figure S3b). A pool of GIGYF1 resides in p-bodies, where it co-localizes with the p-body core component SM-like protein 1 (LSM1) and mRNA-associated factors, such as argonaute 2 (AGO2) and DEAD-Box Helicase 6 (DDX6) (Figure S3c). This localization likely mirrors the function of GIGYF1 as a part of protein complexes that bind to and are inhibitory to mRNA translation. Strikingly, we noticed that wildtype (WT) but not phosphosite-mutated GFP-GIGYF1 was rapidly dispersed from this discrete localization upon treatment of cells with anisomycin (Figure 3e,f), highlighting a functional consequence of stress-induced GIGYF1 phosphorylation.

We conclude that phosphorylation and 14–3–3 binding of GIGYF1 negatively affect GYF domain-dependent protein interactions. We also speculate that additional MK2 phosphorylation events on either GIGYF1, TTP and/or ZNF598 could contribute to this regulation as well.

### 2.3. Multiple Golgi Apparatus-Associated Proteins Are Targets of JNK- and p38-Dependent Phosphorylation

**Figure 4:**
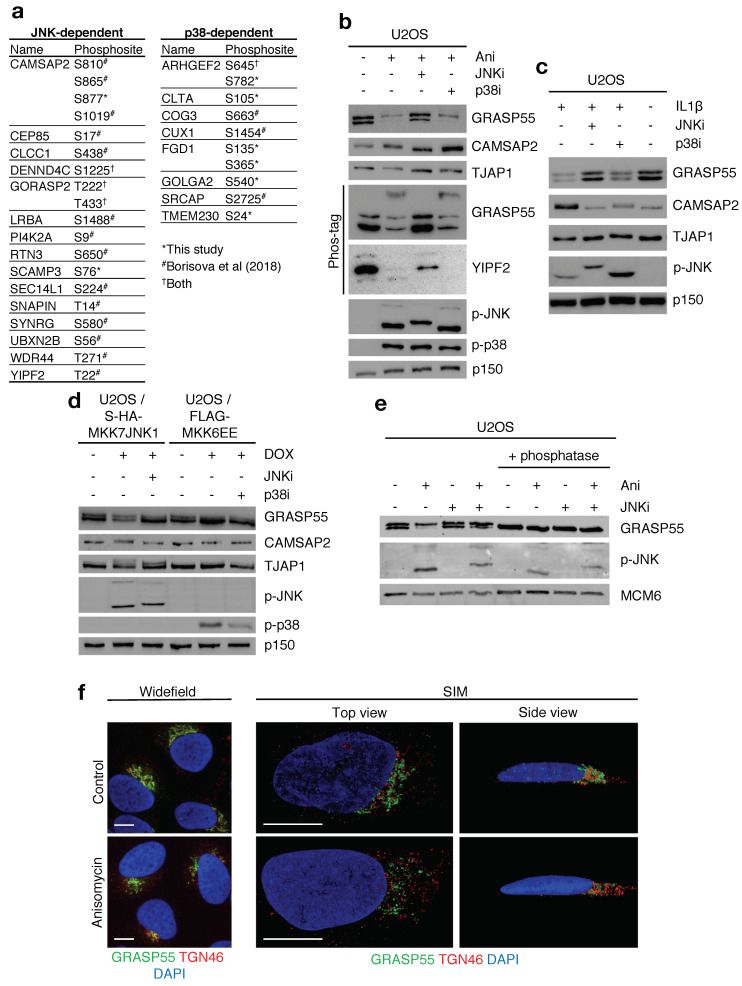
JNK phosphorylation targets in the Golgi apparatus. (**a**). JNK- and p38-dependent phosphorylation sites on Golgi apparatus-resident proteins or Golgi trafficking proteins extracted from Figure 1c, Table S1 and [11]. (**b**). U2OS cells were pre-treated with JNK and p38 inhibitors (JNKi, p38i, 0.5 h) and treated with anisomycin (Ani, 1 h) as indicated. Lysates were separated by SDS-PAGE or phos-tag gel and analyzed by immunoblotting with the indicated antibodies. (**c**). As in (**b**), instead cells were treated with IL1β (1 h). (**d**). U2OS cells conditionally expressing Strep-HA-MKK7JNK1 or FLAG-MKK6EE were induced with doxycycline (DOX), treated and analyzed as in (**b**). (**e**). U2OS cells were pre-treated with JNK inhibitor (0.5 h) and treated with anisomycin (1 h) as indicated. Selected lysates were treated with λ phosphatase and analyzed as in (**b**). (**f**). Representative images of U2OS cells treated with anisomycin (1 h), fixed and immunostained with antibodies against GRASP55 and TGN46 and counter-stained with DAPI. Left: Widefield microscopy. Right: Top and side view of 3D projections acquired by Structured Illumination Microscopy (SIM). Scale bars, 10 μm.

Our data-mining further revealed the presence of a large number of Golgi-resident proteins and proteins associated with Golgi trafficking among the putative novel targets of p38 and JNK phosphorylation. Our phospho-proteomic investigations also strongly suggested that the acute stress-induced phosphorylations that are not mediated by p38 signaling, can largely be attributed to JNK kinases. Therefore, we extended our data mining to a comprehensive published dataset of UV-induced and p38-regulated phosphorylation reactions [11]. We surmised that UV-induced phosphorylation of S/TP motifs refractory to p38 inhibition likely represents direct JNK phosphorylation sites. A combination of these two analyses allowed us to compile a list of high confidence phosphorylation sites on Golgi proteins controlled by JNK or p38 signaling (Figure 4a). Using a combination of standard western blotting and Phos-tag gels, we readily observed JNK-dependent and anisomycin-induced gel retardation of a subset of these proteins; Golgi reassembly-stacking protein of 55kDa (GRASP55/GORASP2), calmodulin regulated spectrin associated protein family member 2 (CAMSAP2), tight junction-associated protein 1 (TJAP1) and YIP1 domain family member 2 (YIPF2) (Figure 4b and Figure S4a). These mobility shifts are strongly indicative of phosphorylation and, importantly, this behavior could also be observed upon activation of JNK by interleukin 1β (IL1β) (Figure 4c). Phospho-shifts were also induced by constitutively active JNK (MKK7-JNK1 fusion) in a manner dependent on JNK kinase activity, but not by activation of endogenous p38 through expression of constitutively active MKK6 (MKK6EE) (Figure 4d).

Among the new JNK targets, the Golgi apparatus-associated protein GRASP55, which links Golgi stacks and ribbons through oligomerization, caught our attention. Interestingly, our analysis revealed that the phosphorylation sites on GRASP55, T222 and T225, which were previously characterized as mitotic phosphorylation sites breaking GRASP55 oligomers [22,23], are modified by JNK upon cellular stress insults (Figure 4a). The two bands with differential gel mobility that we consistently observed, clearly represented the uncharged/unmodified and charged/phosphorylated versions of GRASP55, respectively. Thus, the two bands could be collapsed to a single band by phosphatase treatment (Figure 4e) or mutation of the relevant threonines to uncharged alanines or negatively charged glutamic acids (Figure S4b). These results are in agreement with our phospho-proteomic results and highlight that GRASP55 oligomerization is negatively regulated by JNK activity during cellular stress responses.

### 2.4. MAP Kinase Signaling Restructures the Golgi Apparatus

**Figure 5:**
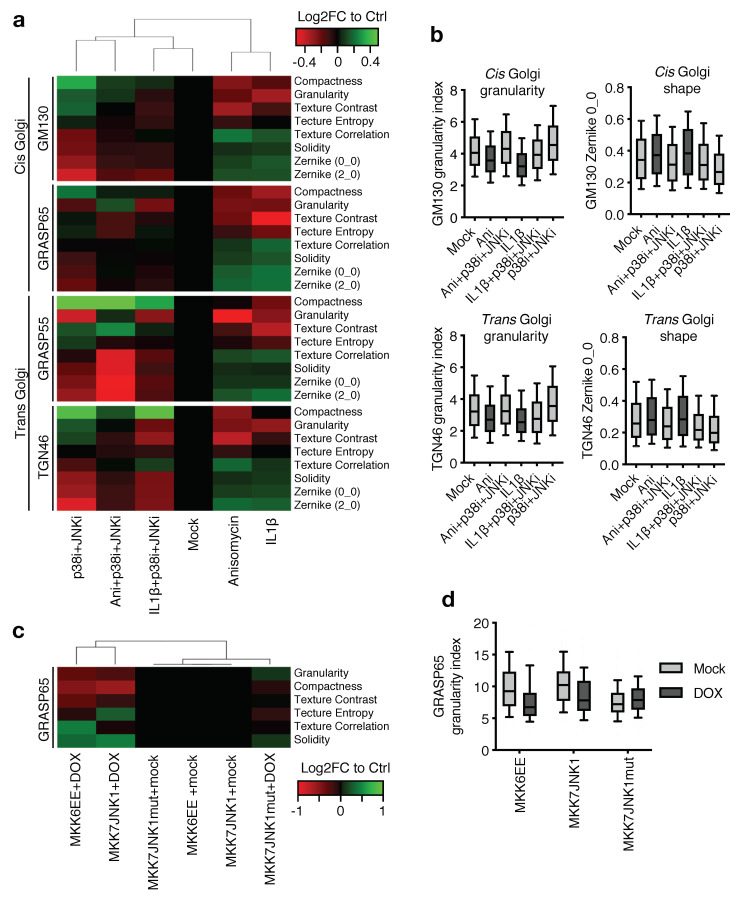
Regulation of Golgi morphology by p38 and JNK. (**a**). Heatmap and horizontal clustering of descriptors of Golgi morphology. U2OS cells were pre-treated with the combination of JNK and p38 inhibitors (JNKi, p38i, 0.5 h) and treated with anisomycin (Ani, 1 h) or IL1β (1 h) as indicated. Cells were fixed, immunostained with antibodies against *cis* Golgi markers GM130, GRASP65 and/or *trans* Golgi markers GRASP55 and TGN46 and images were acquired by high content microscopy. Images were processed and analyzed with CellProfiler software for calculation of the indicated parameters, and are presented as log2-transformed mean fold changes compared to the control. *n* > 1700 cells. (**b**). Box plots of selected non-transformed parameters from (**a**). Dark grey represents conditions with activated p38 and JNK, light grey represents mock or inhibitor-treated samples. Boxes show 25, 50 and 75 percentiles and whiskers show 10 and 90 percentiles. (**c**). As in (**a**), but with U2OS cell lines doxycycline-induced (DOX) for the expression of constitutively active p38 (MKK6EE), JNK (MKK7JNK1) or kinase-dead MKK7JNK1 (MKK7JNK1mut). *n* > 100. (**d**). Box plot of non-transformed granularity index parameters from (**c**). Boxes show 25, 50 and 75 percentiles and whiskers show 10 and 90 percentiles.

To assess the overall impact of stress-induced Golgi protein modification, we turned to fluorescence microscopy, immunostaining for GRASP55 and the *trans*-Golgi marker trans-Golgi network protein 2 (TGN46). In unstressed U2OS cells, both low-resolution widefield microscopy and high-resolution Structured Illumination Microscopy (SIM) revealed a Golgi apparatus with the expected smooth stack morphology and little distance between the two markers. This representation clearly changed with anisomycin treatment to a more granular pattern with greater separation of individual structures (Figure 4f; Figure S5a). These alterations are indicative of partial unstacking and fragmentation of the Golgi apparatus, not unlike the changes that occur during mitosis. To circumvent the inherent cell-to-cell variability in Golgi morphology, we took an unbiased approach to our analysis, combining high-content microscopy of spatially distinct markers and image analysis-based characterization of segmented Golgi signals. With reference to the latter, we took advantage of a previously developed approach [24], where Golgi structures are mathematically described according to their compactness, granularity and texture. We added to this analysis the similarity to Zernike moment-based image shapes. Scan^R based image acquisition of >1700 cells per condition and computational analysis of these features allowed us to make an overall comparison of the differences and similarities between samples. We further submitted all of these data to a hierarchical clustering, which allowed us to make an overall comparison of Golgi morphology upon treatment of cells with anisomycin, IL1β and MAP kinase inhibitors (Figure 5a). The results indicated that both stress- (anisomycin) and inflammatory (IL1β) stimuli are accompanied by large-scale and diffuse structural rearrangements of the Golgi apparatus. Importantly, simultaneous inhibition of JNK and p38 inhibition caused the same treatments to cluster with the control samples (Figure 5a). These effects could also be appreciated by examining some of the individual parameters. Thus, the cellular distributions of granularity indices and Zernike shapes of both the *cis*-Golgi marker *cis*-Golgi matrix protein (GM130) and the *trans*-Golgi marker TGN46 were clearly changed by both anisomycin and IL1β, but reversed by MAP kinase inhibition (Figure 5b). The effects of JNK and p38 activity were clearly additive, in as much that inhibition of both of these kinases caused anisomycin-treated cells to have a stronger resemblance to control cells than each inhibitor alone (Figure S5b,c). Finally, we also used our cell line systems with forced and stimulus-independent activation of JNK or p38 to assess the influence of MAP kinases on Golgi morphology. In this experiment, non-induced cell lines clustered together when analyzed for GRASP65-derived image analysis parameters. Contrarily, induction of either constitutively active MKK7-JNK1 fusion [25] or p38-activating constitutively active MKK6 [26] caused a shift in these parameters and the formation of a distinct cluster (Figure 5c,d). We conclude that JNK- and p38-signaling jointly impact the overall appearance of the Golgi apparatus, and that activation of these kinases induce large-scale structural rearrangements. This dramatic change in morphology likely depends on multiple phosphorylation events on several structural and regulatory Golgi proteins.

### 2.5. JNK and p38 Modulate the Dynamic Properties and Secretory Capacity of the Golgi Apparatus

**Figure 6:**
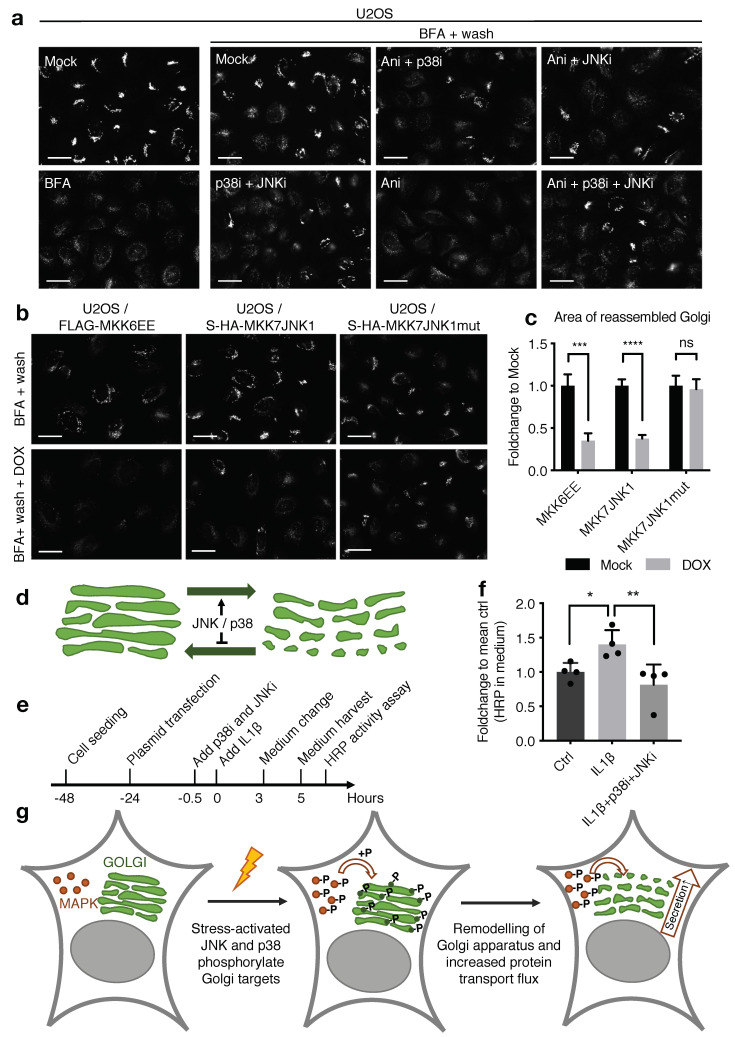
Regulation of Golgi dynamics and protein flux by p38 and JNK. (**a**). To dismantle the Golgi apparatus, U2OS cells were treated with Brefeldin A (BFA–1 h). This was done in the presence of p38 inhibitor, JNK inhibitor (p38i, JNKi, both at time 0) and anisomycin as indicated (Ani, after 0.5 h). BFA was washed out and cells were allowed to recover in the presence of indicated combinations of anisomycin, p38 inhibitor and JNK inhibitor (2 h). Cells were fixed, immunostained with GRASP65 antibodies and counterstained with DAPI. (**b**). As in (**a**), but with U2OS cell lines doxycycline-induced (DOX) for the expression of constitutively active p38 (MKK6EE) or JNK (MKK7JNK1) as indicated. Cells were treated with BFA (1 h) and fixed after wash-out and recovery (1 h). (**c**). Quantification of (**b**). The area of reassembled Golgi was measured with CellProfiler. The graph shows the fold change to own mock-treated control and error bars represent the standard error of mean. *n* > 100. ***, *p* < 0.001 and ****, *p* < 0.0001 using a two-way ANOVA with Benjamini, Krieger and Yekutieli’s correction for multiple comparison. (**d**). Activated p38 and JNK additively mediate disassembly and prevent reassembly of the Golgi apparatus. (**e**). Schematic of exocytosis assay. U2OS cells were transfected with a plasmid encoding HRP enzyme modified to pass through the secretory system. Exocytic flux was evaluated by measuring HRP activity secreted to the medium 3–5 h after IL1β treatment. (**f**). Quantification of (**e**). Cells were treated with IL1β and p38 + JNK inhibitors (p38i, JNKi) as indicated. The graph shows the fold change to mean control and error bars represent the standard deviation. *n* = 4, ns, no significance; *, *p* < 0.05 and **, *p* < 0.01 in one-way ANOVA with Benjamini, Krieger and Yekutieli’s correction for multiple comparison. (**g**). Model of the regulation of Golgi morphology and protein transport by p38 and JNK. All scale bars, 20 μm.

The Golgi apparatus is a remarkably dynamic organelle. This property is important for normal physiology and is often dysregulated in pathology, and allows the Golgi apparatus to assume a host of cell type-specific forms. As a testament to its dynamic properties, the Golgi apparatus is rapidly disrupted by Brefeldin A (BFA), a fungal inhibitor of COPI-mediated membrane transport. Conversely, Golgi stacks are quickly re-formed upon the removal of BFA. We noticed that Golgi reappearance after BFA wash-off was dramatically inhibited by treatment with anisomycin (Figure 6a), which we took as an indication that MAPK signaling not only changes Golgi morphology but also alters its dynamic properties. Of note, puromycin, another inhibitor of ribosome function that does not cause robust activation of MAP kinases, did not show this effect (Figure S5d). The negative effect of anisomycin on the re-formation of the Golgi apparatus appeared to be caused by JNK and p38 signaling, as inhibitors of these kinases abrogated this effect in an additive manner (Figure 6a). These findings were corroborated in our cell lines with genetic and stimulus-independent activation of JNK and p38 signaling (Figure 6b,c). Combined, our imaging data suggests that MAP kinase signaling negatively impacts on the dynamic properties of the Golgi apparatus, which may be the underlying cause of the morphological alterations that we observe (Figure 4f, Figure 6d and Figure S5a). A Golgi apparatus with a less stack-like and more granular structure has been associated with accelerated protein transport. Reducing the time proteins spend in these compartments leaves less time for Golgi-associated protein modifications, such as the formation of elaborate sugar structures [27,28]. Such changes have potential physiological consequences, as they impact on the structures of proteins presented on the cell surface and secreted to the extracellular milieu. Indeed, we found that the bulk release of a model exocytosis reporter (HRP enzyme [29]) was increased upon IL1β treatment of cells, and this effect was dependent on MAP kinase activation (Figure 6e,f). Collectively, our results suggest that MAP kinase signaling dramatically impacts on the morphology and dynamic properties of the Golgi apparatus. These changes, in turn, have a direct impact on the secretory capacity of cells.

## 3. Discussion

In this study, we have performed an unbiased phospho-proteomics screen for new substrates of the MAP kinases p38 and JNK, resulting in the identification of >450 regulated phosphorylation sites. Our work has uncovered that the abundance of JNK targets is in the same magnitude as p38-dependent targets. Our analysis also suggests that some of the previously published screens combining cyclin-dependent kinase-inhibiting stress stimuli (such as UV-irradiation and anisomycin) with p38 inhibitors can be re-mined for potential JNK targets by filtering for S/TP sites that are stress-induced and p38-independent. Especially among the JNK-regulated sites, we noticed that many were previously annotated as mitotic CDK phosphorylation sites (Figure 1d), suggesting that stress conditions and mitosis have some regulatory aspects in common. While we do not understand the biological ramifications of this, it is worth noting that many CDK phosphorylation events in mitosis serve to silence or inhibit normal cellular functions that could be deleterious during the process of cell division. Acute stress and inflammatory stimuli may pose similar demands on cells for prioritization, and MAP kinases could be the masterminds of such regulation.

Among the many MAP kinase targets we mapped, were the GYF-domain containing proteins GIGYF1 and 2. These proteins employ their GYF domain to link poly-proline-containing and RNA-binding proteins such as ZNF598 and TTP to the non-productive mRNA cap-binding protein 4EHP to inhibit translation of specific transcripts [20,21,30]. Increasing evidence suggests that GIGYF proteins also directly recruit destabilizing activities to 4EHP-bound mRNAs [31]. These complexes have been implicated in a variety of posttranscriptional regulatory processes, such as Ribosome-associated Quality Control (RQC) and inflammatory signaling [21,32,33,34]. Through binding to ZNF598 and TTP, GIGYFs exert negative control over a wide spectrum of translation reactions [30]. Our results show that GIGYF proteins are phosphorylated by the p38-downstream kinase MK2 at two sites flanking the GYF domain. These reactions allow for interaction with 14–3–3 proteins, negatively impacting on the ability of the GYF domain to engage in protein-protein interactions. Through such regulation, 4EHP-GIGYF1/2 will be ejected from at least a subset of its normal mRNA-containing complexes. As a likely consequence of this, we observed that an MK2-resistant phosphorylation mutant of GIGYF1 was not displaced from mRNA-containing p-bodies in response to p38-activating anisomycin treatment. Of note, there are several reports on association of GIGYFs with other RNA metabolic factors independently of its GYF domain [31,35], and GIGYF2 has even been suggested to bind directly to RNA [36]. The role of GIGYF in translational regulation, and the regulation of these functions by stress kinases, may, thus, be multifactorial and complex.

The best-established role of ZNF598 is as a molecular sensor of collided ribosomes, a function required for ribosome rescue in the RQC pathway [37,38]. Mutation of both GIGYF2 and RQC components, such as Listerin/LTN1 and nuclear export mediator factor (NEMF), cause neurological disorders in mice [39,40]. In humans, heterozygous mutation of the GIGYF2 gene has been linked to familial Parkinson’s disease [41]. There appears to be a direct link to GIGYF2′s ability to interact with RNA-binding factors, as mutation of the GYF domain was linked to late-onset Parkinson’s disease in a Spanish family [42]. Recently, the larger 4EHP-GIGYF1/2-ZNF598 complex has been connected to these functions, acting to prevent loading of new ribosomes on blocked transcripts. This role is seemingly independent of ZNF598′s ability to ubiquitinate ribosomal proteins and mediate dissolution of stalled and collided ribosomes [32,33,34]. The MAPKKK leucine zipper- and sterile alpha motif-containing kinase α (ZAKα) is also activated by ribosome collisions [43] to mediate activation of p38 [44], a signaling pathway generally referred to as the Ribotoxic Stress Response (RSR) [45]. Our finding that GIGYF proteins are substrates for p38-mediated phosphorylation and 14–3–3 binding suggests that there is crosstalk between signaling pathways activated by the same signal of translational arrest. It is at present not clear how RSR-induced GIGYF modification impacts RQC-mediated impairment of ribosome loading, and this will be an important issue for future investigation. The RSR pathway is not conserved in yeast, but also in this organism it was reported that there is considerable cross-regulation/competition between pathways activated by collided ribosomes [46].

Mining of our dataset revealed several p38- and JNK-dependent phosphorylation sites on Golgi apparatus-resident proteins, and this list could be expanded considerably by re-mining of a published dataset (Figure 4a). Among these were GRASP55, which, similar to its homolog GRASP65, is a Golgi membrane-attached protein, and through its oligomerization, it is responsible for the stacking and linking of ribbons of Golgi cisternae in interphase cells. Although not completely spatially separated, GRASP55 largely performs this task in *medial*- and *trans*-Golgi stacks, while Golgi reassembly-stacking protein of 65 kDa (GRASP65) function is confined to *cis*-Golgi stacks. Upon mitosis, phosphorylation of the serine-proline-rich (SPR) domains break this oligomerization, which is critical for Golgi unstacking, vesiculation and partitioning to the daughter cells [47]. We were intrigued by the possibility that the Golgi apparatus could be a prominent target for stress-induced MAP kinase signaling, for which we only found limited evidence in the literature. One report describes how JNK signaling contributes to Golgi fragmentation prior to mitosis [48]. Intriguingly, both acute stress (anisomycin) and an inflammatory stimulus (IL1β) rapidly changed the overall appearance of the Golgi apparatus (Figure 4f, Figure 5a,b and Figure 6d). The Golgi network is a complicated macromolecular structure with a highly cell-heterogenic representation, the morphological characterization of which is not trivial. Several studies have attempted to devise computational tools for the determination of structural alterations to this cellular compartment. We successfully adapted one such approach, using the open source image analysis software CellProfiler [24]. This allowed us to quantify signal-based and structure-derived parameters, such as compactness, granularity and Zernike shapes. Systematic determination of these parameters in individual cells was used to quantify structure-derived features. This allowed us to computationally cluster and separate averaged Golgi signatures from differentially treated samples. An overall visual inspection of our images gave us the impression that cell stress and inflammatory stimulation resulted in a more granular and disorganized Golgi structure (Figure 4f and Figure S5a). This is supported in both heatmaps of all parameters (Figure 5a and Figure S5b) and box plots of individual parameters from our computational analyses (Figure 5b and Figure S5c). Inhibitors of p38 and JNK reversed these changes in an additive fashion, suggesting that both kinases are required to manifest the observed structural regulation of the Golgi apparatus. In support, forced, yet artificial, activation of either the p38 or the JNK pathway brought about similar structural changes (Figure 5c,d). In addition to, or possibly as a consequence of, the above, cell stress also changed the dynamic properties of the Golgi apparatus in a p38- and JNK-dependent manner (Figure 6a–c). This manifested as an inability to re-form the Golgi apparatus upon release from BFA-induced disruption of the structure. Given the breadth of Golgi-relevant p38 and JNK substrates we identified (Figure 4a), it is unlikely that it will be feasible to pinpoint single or combined phosphorylation events that mediate the changes described above. The observed restructuring may well result from the sum of several small-scale perturbations of protein structures.

A lower degree of compactness and higher degree of fragmentation of the Golgi apparatus has been correlated with higher protein flux through the secretory pathway [27,28], and this is indeed what we observed for an artificial exocytic substrate (Figure 6e,f). As less time spent in the Golgi network also reduces the time afforded for glycosylation reactions, such fast-tracked proteins have also been shown to be under-glycosylated [27,28]. Thus, we hypothesize that MAP kinase regulation of the structural and dynamical properties of the Golgi apparatus could have important biological ramifications (Figure 6g). This is likely relevant to inflammatory responses that depend on production and secretion of cytokines. Cytokine production is already regulated by p38 and JNK activity on several levels, including gene transcription and stabilization and translation of the resulting mRNA [4]. We propose that modulation of the secretory system could be yet another focus point for regulatory intervention, all with the purpose of ensuring rapid and robust secretion of immune system-modulating factors such as cytokines.

In conclusion, our work highlights the pervasive role of p38 and JNK signaling during inflammatory and cell stress responses, and identifies the Golgi apparatus as a hitherto unappreciated target of regulation by these kinases.

## 4. Materials and Methods

### 4.1. Plasmids and siRNA

pcDNA4/TO-GFP-GIGYF1 (Addgene plasmid #141188), pcDNA4/TO-GFP-GIGYF2 (Addgene plasmid #141189), pcDNA4/TO-GFP-TTP (Addgene plasmid #141190), pGEX2TK-*p*-GST-14–3–3-epsilon and pGEX2TK-*p*-GST-14–3–3-zeta were previously published [21,49]. Plasmids encoding GFP, GFP-MK2 and GFP-MK2 K79R were also previously described [50]. GIGYF1 truncations were PCR-cloned into pEGFP-C1 using XhoI and EcoRI restriction sites. All mutations in GIGYF1 were introduced by site-directed mutagenesis using KOD DNA polymerase (Merck Millipore, Darmstadt, Germany) according to the manufacturer’s instructions. pFLAG-4EHP was a gift from Dong-Er Zhang (Addgene plasmid #17342). pcDNA3-FLAG-MKK6 S207E/S211E, MKK7B2JNK1a1 and MKK7B2JNK1a1APF were gifts from Roger Davis (Addgene plasmids #13518, #19726, #19730). These constructs were PCR-cloned into pcDNA4/TO/Strep-HA using NotI and XbaI. Rat GRASP55wt-GFP, GRASP55T222A–T225A–S245A-GFP and GRASP55 T222E-T225E-GFP (a gift from Dr. Yanzhuang Wang, the University of Michigan, USA) were PCR-cloned into pcDNA4/TO/Strep-HA using EcoRV and NotI. SS-HRP-Flag was a gift from Vivek Malhotra, Centro de Regulacion Genomica, Spain. All plasmids were verified by sequencing. Plasmid transfections were carried out using Fugene6 (Promega, Madison, WI, USA), as described in the manufacturer’s protocol. siRNA target sequences used in this paper include: siGIGYF1 #1 (GUUAGGAGGCUGAAAGAAAUU), siGIGYF1 #2 (GGGAAGAGGAAGAGCGAAA) and siCTRL (GGGAUACCUAGACGUUCUA). siRNA transfections were carried out using RNAiMAX (Life Technologies, Carlsbad, CA, USA) as described in the manufacturer’s protocol.

### 4.2. Cell Culture and Treatment

Human U2OS osteosarcoma cells (ATCC), HEK293 embryonic kidney cells (ATCC), and hTERT-immortalized RPE1 epithelial cells (ATCC) and MK2/MK3 DKO MEF [50] were cultured in Dulbecco’s Modified Eagle’s Medium (DMEM, Biowest, Nuaillé, France) with L-glutamine medium. Medium was supplemented with 10% fetal bovine serum (FBS, Biowest) and 1% penicillin and streptomycin. Cells were maintained at 37 °C in a humidified 5% CO_2_ cell incubator. Stable cell lines were generated by transfecting cells with a plasmid containing resistance gene and the gene of interest. Cells were subsequently cultured for weeks in medium containing appropriate antibiotic selection (Zeocin (0.2 µg/mL, Thermo Fisher, Waltham, MA, USA), Blasticidin S (5 µg/mL, Thermo Fisher), G418 (0.4 mg/mL, Thermo Fisher). Individual cell clones were screened for gene expression by immunofluorescence and western blotting. To generate inducible stable cell lines, pcDNA4/TO-constructs carrying the gene of interest were co-transfected with pcDNA6/TO (Thermo Fisher) in a ratio of 1:4. The following chemicals and inhibitors were used in this paper: Anisomycin (2 µg/mL), cycloheximide (10 µM), recombinant human Interleukin 1β/IL1β) (2 ng/mL) (Peprotech, Stockholm, Sweden), JNK-IN-8 (10 μM, 5 μM overnight) (Sigma, St Louis, MO, USA), SB203580 p38 inhibitor (10 μM, Cell Signaling, Danvers, MA, USA), MK2 inhibitor PF3644022 (10 µM, Sigma), Brefeldin-A (5 μg/mL) and doxycycline (1 μg/mL). UV irradiation (50 J/m^2^) was delivered in a BS-02 irradiation chamber equipped with 254 nm bulbs (Gröbel Elektronik, Germany).

### 4.3. Proteomics

SILAC and TMT labelling, phospho-enrichment and mass spectrometry were carried out as described [51].

### 4.4. Raw Data Processing

All phospho-proteomics raw files were processed with MaxQuant [52] v1.6.14.0 using the integrated Andromeda Search engine [53]. Data were searched against the human Uniprot Reference Proteome (2020, October release). TMT10plex was specified as quantification method. Trypsin was specified as enzyme, cleaving after all lysine and arginine residues and allowing up to two missed cleavages. Carbamidomethylation of cysteine was specified as fixed modification and protein *n*-terminal acetylation, oxidation of methionine and phosphorylation of serine, threonine and tyrosine were considered variable modifications with a total of three variable modifications per peptide. “Maximum peptide mass” was set to 7500 Da, the “modified peptide minimum score” and “unmodified peptide minimum score” were set to 25 and everything else was set to the default values, including the false discovery rate limit of 1% on both the peptide and protein levels.

### 4.5. Data Analysis

Phosphorylation site intensities were retrieved from Phospho(STY).txt table. “Reverse” and “Potential contaminants” were removed for further analysis. Data were collapsed to sites in Perseus (v1.6.15.0). Data were log2 transformed and phosphorylation sites not quantified in all ten TMT channels were filtered out. Data were imported into Prostar (v1.18.4) [54] for normalization (method Loess) and differential expression analysis using limma. Statistically significant differentially regulated sites were defined for each pairwise comparison as those with an absolute fold change >0.5 (FDR 5%). JNK- and p38-dependent phospho-sites were defined as those that were significantly increased after anisomycin treatment (Anisomycin vs. Control), and those that decreased after the addition of inhibitors (Anisomycin+p38i vs. Anisomycin or Anisomycin+JNKi vs. Anisomycin). Filled logo motifs were generated in iceLogo [55] using only class I sites (PEP > 0.75, Score > 5). Functional annotation was performed at gene level in String (v11) using the whole human proteome as a reference background. Calculation of phosphorylation site occupancy was carried out as described [51].

### 4.6. Western Blotting

Cells were lysed for whole cell lysates using EBC buffer (50 mM Tris, pH 7.5, 150 mM NaCl, 1mM EDTA, 0.5% NP-40, protease and phosphatase inhibitors). For phosphatase treatment, cells were lysed in EBC buffer without EDTA supplemented with 1 mM MnCl_2_ and then incubated with lambda protein phosphatase (P0753L, NEB, Ipswich, MA, USA) at 30 °C for 30 min. Pull-downs of tagged proteins were carried out using GFP Trap magnetic agarose (Chromotek, Planegg, Germany) or Glutathione sepharose 4B(Sigma) for 1 h at 4 °C as previously described [49]. GIGYF1 immunoprecipitations were performed with GIGYF1 antibody (121784, Abcam, Cambridge, UK) coupled to Protein A Sepharose beads (Thermo Fisher). All immunoprecipitations were carried out in low-salt EBC lysis buffer (150 mM NaCl; 50 mM Tris, pH 7.5; 1 mM EDTA; 0.5% NP40). Samples were mixed with Laemmli sample buffer and boiled before being resolved on SDS-PAGE and transferred to nitrocellulose membranes. Membranes were blocked in PBS-T + 5% milk and then probed with the following antibodies overnight: Phospho RxxS*/T* (Cell signaling, 9614, rabbit), GIGYF1 (Abcam, ab121784 and ab121646, rabbit), MK2 (Cell signaling, 3042, rabbit), CHK1 (S345, Cell signaling, 2358, rabbit), 14–3–3 (pan, Cell signaling, 8312, rabbit), GST (Santa Cruz, discontinued, sc459, rabbit), TTP (Sigma, T5327, rabbit), ZNF598 (Abcam, ab111698, rabbit and/or Sigma, HPA041760, rabbit), FLAG (Sigma, F1804, rabbit and/or Sigma, F1804, mouse) or 4EHP (Cell signaling, 6916, rabbit), GRASP55 (Abcam, Ab211532, mouse), CAMSAP2 (Novus, Abingdon, UK, NCP1–21402, rabbit), TJAP1 (Sigma, HPA030165, rabbit), GM130 (BD biosciences, Franklin Lakes, NJ, USA Clone 35, mouse), YIPF2 (Santa Cruz, sc-398530, mouse), *p*-JNK (Cell signaling, 9255, mouse), *p*-p38 (T180/Y182, Cell signaling, 9216, mouse), p150 (BD biosciences, 610473, mouse), MCM6 (Santa Cruz, goat), HA (Santa Cruz, sc-7392, mouse) and GFP (Torrey Pines, Secaucus, NJ, USA TP401, rabbit). The next day, membranes were washed in PBS-T and incubated for 1 h with Goat Anti-Rabbit or Goat Anti-Mouse IgG Antibody (H+L) Peroxidase (Vector laboratories, Burlingame, CA, USA). PBS-T washing was repeated and antibody signal was visualized by chemiluminescence (Clarity Western ECL substrate, Bio-Rad, Hercules, CA, USA) with a Bio-Rad Chemidoc imaging system.

### 4.7. Phos-Tag Gel

Phos-tag gel experiments were carried out as described for western blotting but with the following exceptions. Samples for phos-tag gels were lysed using EBC buffer without EDTA. Lysates were resolved on a gel containing 25 μM Phos-tag. The gel was washed three times for 15 min in transfer buffer supplemented with 10 mM EDTA and transferred on a PDVF membrane in transfer buffer supplemented with 0.1% SDS and 1 mM EDTA. Membrane incubations were carried out in TBS-T + 5% BSA and washes in TBS-T.

### 4.8. In Vitro Kinase Assay

GFP-tagged GIGYF1 was immunopurified from transfected U2OS cells lysed in high-salt EBC buffer (50 mM Tris, pH 7.5; 500 mM NaCl; 1 mM EDTA; 0.5% NP40; 1 mM dithiothreitol (DTT)). Beads were washed extensively with lysis buffer and once with kinase buffer (25 mM HEPES, pH 7.2; 25 mM MgCl; 2 mM DTT). Reactions were initiated by adding 450 ng recombinant MK2 (Abcam) and 25 mM ATP spiked with γ-P^32^-ATP (10 mCi, Perkin Elmer) to each sample. Indicated samples were supplemented with 100 ng recombinant HSP27 (Prospec, Rehovot, Israel) to serve as positive control for MK2 activity. Reactions were incubated for 30 min at 30 °C with gentle shaking and terminated by adding Laemmli buffer and boiling the samples at 95 °C for 10 min. Samples were then run on SDS-PAGE and vacuum-dried on a Whatman filter paper (Sigma). Relative phosphorylation was assayed by autoradiography.

### 4.9. Immunofluorescence Microscopy

For immunofluorescence microscopy, cells were grown on coverslips and treated as indicated. Cells were fixed for 10 min in paraformaldehyde (Lilys væske, Ampliqon, Odense, Denmark) and permeabilized with 0.2% TritonX-100 for 5 min. For pre-extraction, cells were incubated for 1 min with 0.2% TritonX-100 prior to fixation. Primary antibody incubation was carried out at RT for 1 h using the following antibodies diluted in DMEM: LSM1 (Sigma, HPA024601, rabbit), Ago2 (Merck Millipore, MABE253, Rat), DDX6 (Bethyl, Montgomery, TX, USA, A300–5460A, rabbit), GRASP55 (Abcam, Ab211532, mouse), GRASP65 (Abcam, Ab174834, rabbit), TGN46 (Abcam, Ab50595, rabbit) and GM130 (BD biosciences, Clone 35, mouse). Cells were washed with PBS and incubated for 30 min at RT with the following secondary antibody diluted in DMEM: Alexa Fluor 488 goat anti-mouse (A11001), 568 goat anti-mouse (A11004), 488 goat anti-rabbit (A11008), 568 goat anti-rabbit (A11011) and 568 goat anti-rat (A11077). Finally, cells were washed with PBS, rinsed in water and mounted on glass slides with Vectashield containing Dapi (Vectashield, Vector laboratories, H-1200).

High content microscopy images were acquired using Olympus IX-83 wide-field microscope controlled by ScanR acquisition.

For 3D-SIM, cells were treated as indicated above with the following exceptions: Cells were grown on 18 mm × 18 mm #1.5 coverslips. Coverslips were blocked for 30 min using 2% BSA prior to primary antibody incubation. Secondary antibody incubation was supplemented with DAPI (1 μg/mL). Washes were performed with 0.2% tween in PBS and coverslips were mounted with H-1000 Vectashield. Imaging was performed on a Zeiss ELYRA PS.1 Super Resolution Microscope with Plan-Apochromat 63×/1.4 oil objective and 405, 488 and 561 HR Diode lasers (50, 200 and 200 mW). Acquisition, reconstruction, channel alignments and quality control were performed as described earlier in [56]. Around 50 (512 × 512pixel) images were taken per z-stack (three angles and five phases) with a typical Z-stack height of 6 μm and a z-stack difference on 110 nm, *n* > 10 replicates (13 ani and 15 ctrl).

### 4.10. Image Analysis

Image analysis of Golgi morphology was performed using CellProfiler (2.1.1). For heatmaps, log2 fold change to control was calculated for the mean of each measurement.

### 4.11. Statistical Analysis

Statistical analyses were performed in GraphPad Prism 8.

### 4.12. Golgi Reassembly Assay

Cells were treated for with 5 μg/mL Brefeldin A (BFA, Cell Signaling) for 1 h to disrupt the Golgi structure. Following this, cells were washed twice with PBS and incubated in medium without BFA for 2 h before being fixed. Additional treatments were added as indicated.

### 4.13. Exocytosis Assay

Culture medium from cells stably expressing SS-HRP-FLAG was centrifuged at 300× *g* and supernatant was mixed with o-Dianisidine (Sigma D9154) and peroxide. The resulting brown product was used as a measurement of HRP content and quantified by measuring absorbance at 455 nm using a spectrophotometer.

## Supplementary Materials

The following are available online at https://www.mdpi.com/article/10.3390/ijms22179595/s1.
