# Supplementary material for: Regulation of the Golgi Apparatus by p38 and JNK Kinases during Cellular Stress Responses
Source: Int J Mol Sci. 2021 Sep 4;22(17):9595. doi: 10.3390/ijms22179595 (PMC8431686; doi:10.3390/ijms22179595)
Supplement: Supplementary file 1 [file ijms-22-09595-s001.zip › Supplemental figures.pdf]

Nordgaard et al, Figure S1

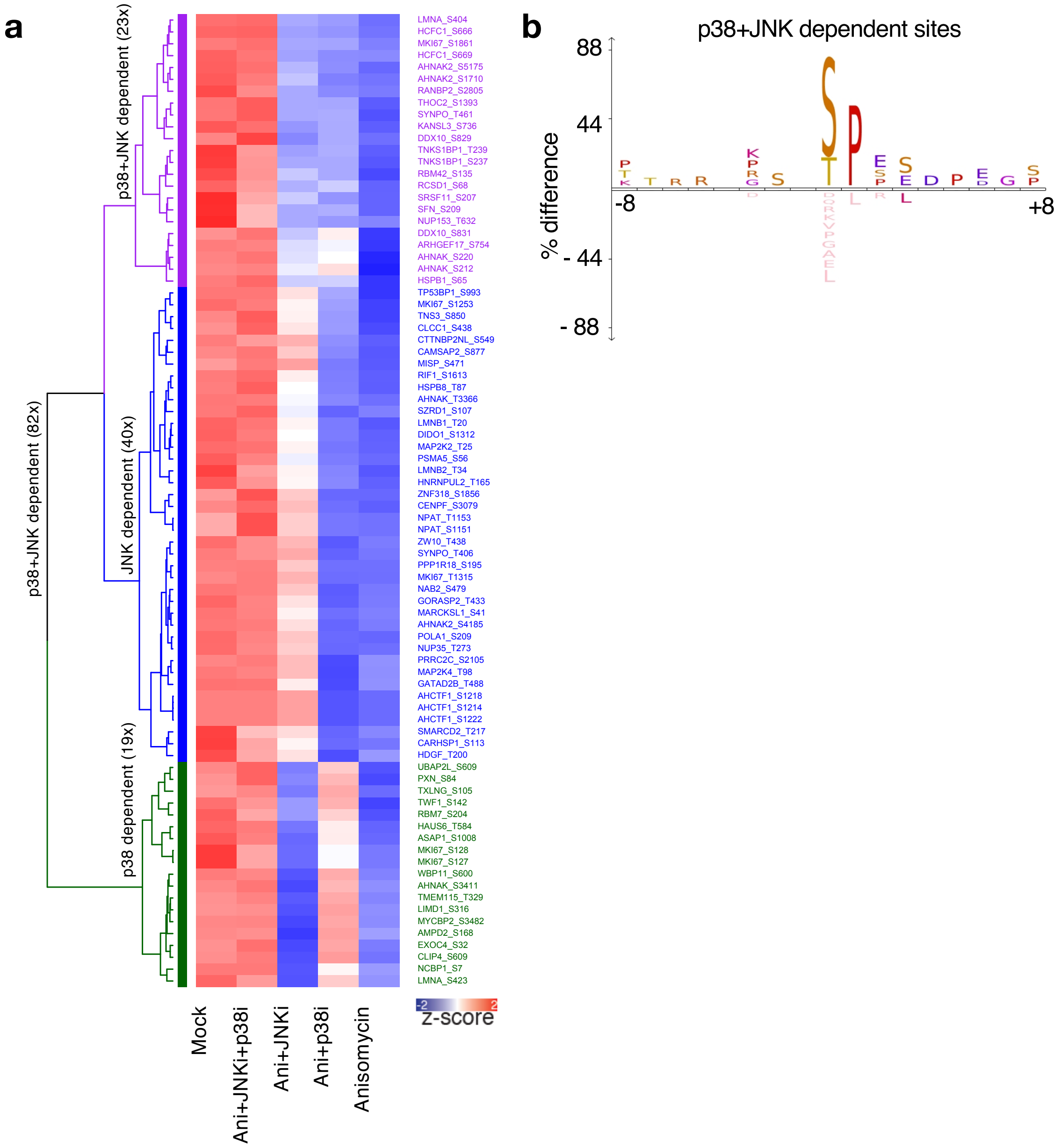

Figure S1.

Clustering and phosphorylation motif of p38+JNK dependent phosphosites.

a. Heatmap with independent clustering of the 82 ‘purple’ anisomycin upregulated and p38+JNK dependent phospho-sites from figure 1c,f. Intensity is represented as z-score. Green, p38 dependent cluster; blue, JNK dependent cluster and purple, p38+JNK dependent cluster. **b.** Sequence motif of p38+JNK dependent phosphorylation sites from figure 1c,f.

Nordgaard et al, Figure S2

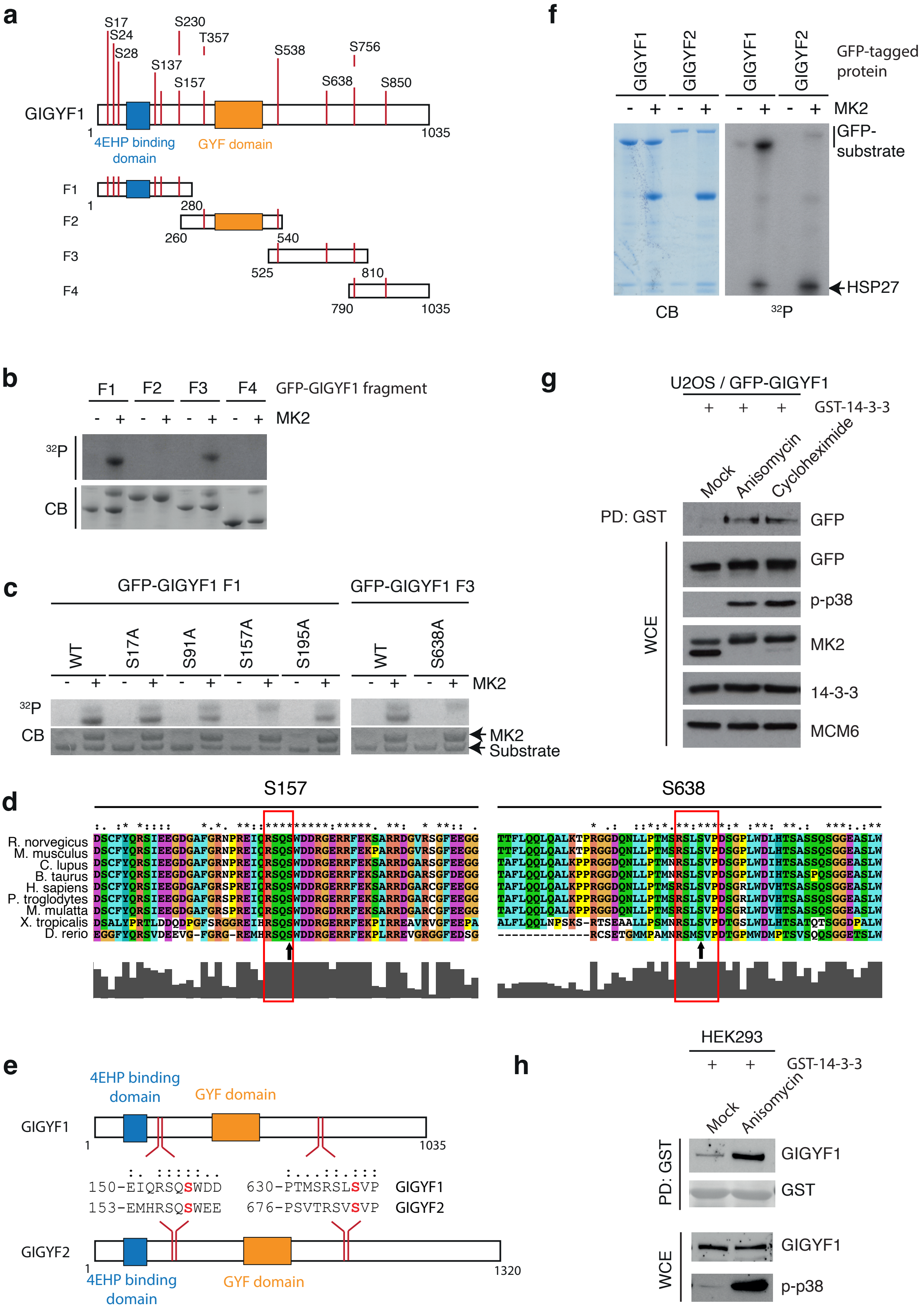

Figure S2.

MK2 phosphorylation of GIGYF1 and GIGYF2.

a. Schematic of overlapping GFP-tagged GIGYF1 fragments (F1-F4) used for *in vitro* kinase assays. **b.** Fragments from (a) were expressed in HEK293 cells, purified and used as substrates for phosphorylation by recombinant MK2. Reactions were resolved by SDS-PAGE and analyzed by autoradiography (^{32}P) and coomassie blue (CB) staining. **c.** As in (b) but with alanine-substituted versions of F1 and F3 fragments from (a). **d.** Multiple sequence alignment of GIGYF1 from multiple species using ClustalX 2.1. Red boxes frame the conserved phosphorylation motifs and 14-3-3 binding consensus sequence from figure 2g. Arrows indicate the phosphoserines. **e.** Alignment of homologous MK2 phosphorylation sites in GIGYF1 and GIGYF2. Phosphorylated residues are highlighted in red. **f.** As in (b), except that full-length GFP-GIGYF1 and GFP-GIGYF2 were used as substrates. **g.** U2OS cells stably transfected with GFP-GIGYF1 were treated with anisomycin (1 h) or cycloheximide (1 h) as indicated. Lysates were incubated with recombinant GST-14-3-3 protein. GST pull-down material (PD: GST) and whole cell extracts (WCE) were analyzed by immunoblotting with the indicated antibodies. **h.** HEK293 cells were treated with anisomycin (1 h). Lysates were incubated with recombinant GST-14-3-3 protein and analyzed as in (g).

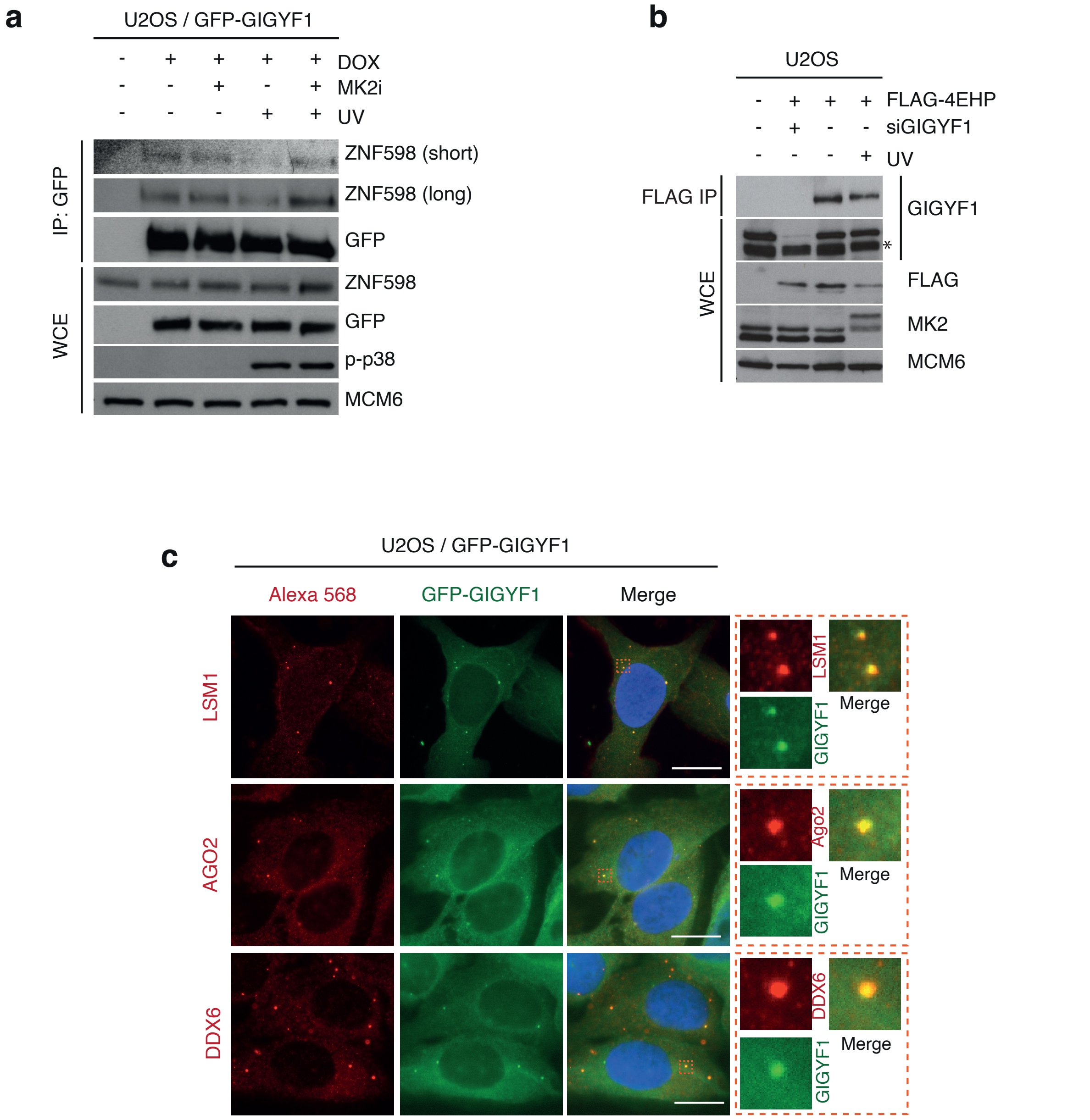

Figure S3.

Mapping of MK2 phosphorylation sites and p-body localization of GIGYF1.

a. U2OS cells conditionally expressing GFP-GIGYF1 were induced by doxycycline (DOX), pre-treated with MK2 inhibitor (0.5 h) and UV-irradiated (50 J/m^2, 1 h recovery). GFP pull-down material (IP: GFP) and whole cell extracts (WCE) were analyzed by immunoblotting with the

indicated antibodies. **b.** U2OS cells were transfected with FLAG-4EHP plasmid and GIGYF1 siRNA and UV-irradiated (50 J/m^2, 1 h recovery) as indicated. FLAG-tag immunoprecipitated material (IP: FLAG) and whole cell extracts (WCE) were analyzed by immunoblotting with the indicated antibodies. Asterisk denotes an unspecific band. **c.** Cells from (a) were fixed and immunostained with antibodies against LSM1, AGO2 or DDX6 and counter-stained with DAPI. Inserts show co-localization between GFP-GIGYF1 and p-body markers. Scale bars, 10 μm.

Nordgaard et al, Figure S4

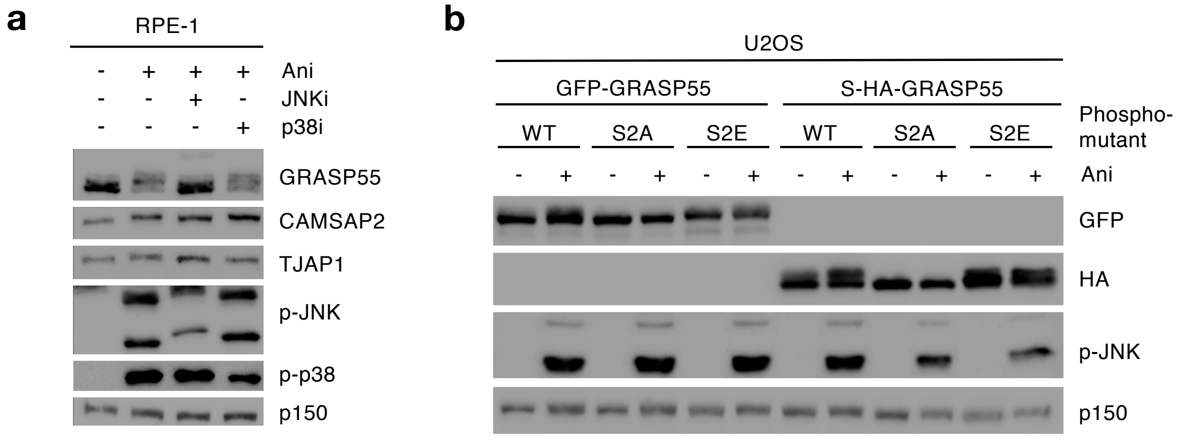

Figure S4.

Validation of T222 and T225 in GRASP55 as JNK phosphorylation sites.

a. RPE-1 cells were pre-treated with JNK and p38 inhibitors (JNKi, p38i, 0.5 h) and treated with anisomycin (Ani, 1 h) as indicated. Lysates were analyzed by immunoblotting with the indicated antibodies. **b.** U2OS cells transiently transfected with wildtype (WT), non-phosphorylatable (S2A) or phospho-mimicking (S2E) versions of GFP-GRASP55 or Strep-HA-GRASP55 were treated with anisomycin (1 h) as indicated. Lysates were analyzed by immunoblotting with the indicated antibodies.

Nordgaard et al, Figure S5

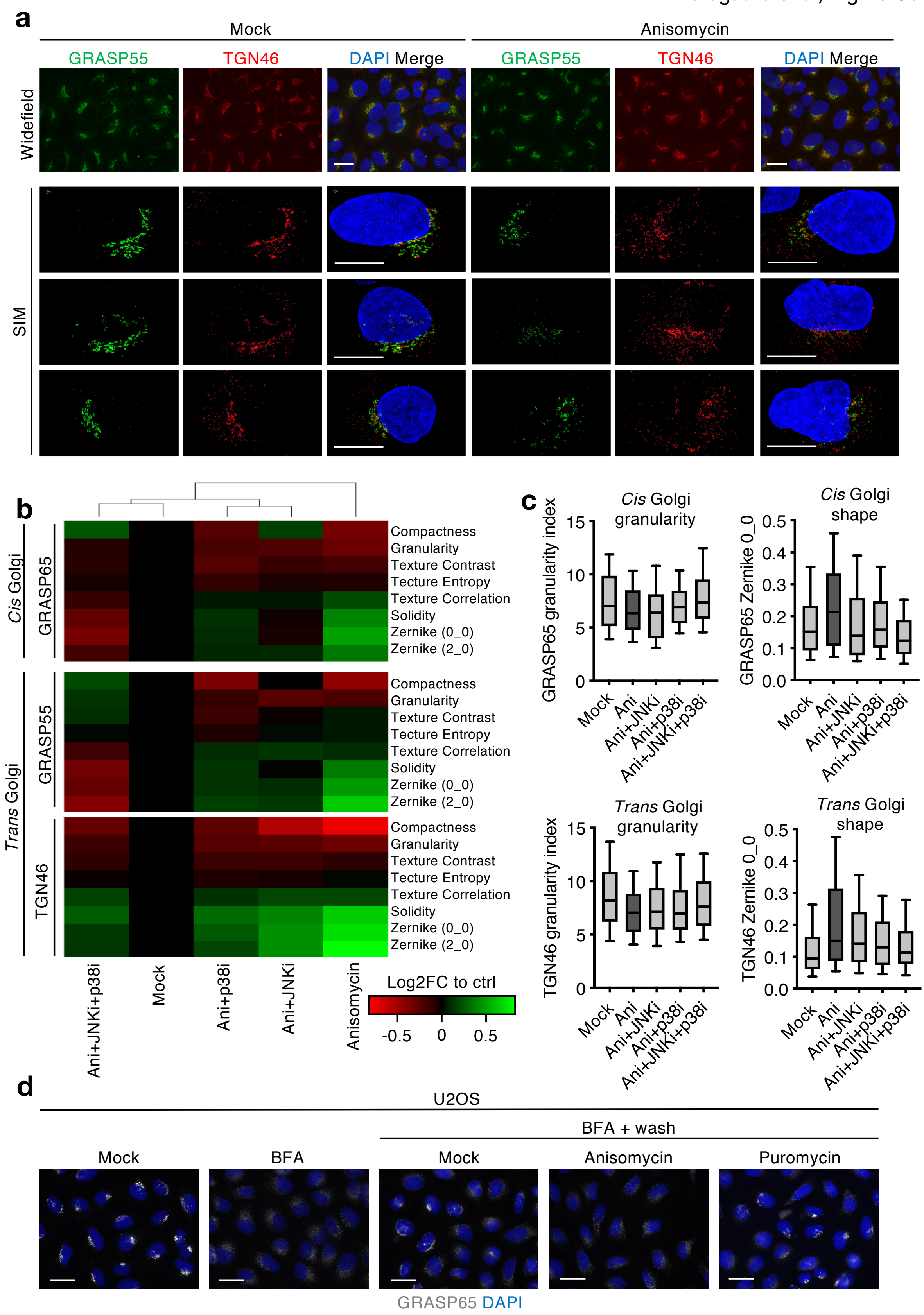

Figure S5.

Relative contribution of p38 and JNK kinases to Golgi regulation.

a. U2OS cells were treated with anisomycin (1 h), fixed and immunostained with antibodies against GRASP55 and TGN46 and counter-stained with DAPI. Top row: Widefield microscopy. Lower three rows: 3D projections acquired by Structured Illumination Microscopy (SIM). Scale bars, 10 μm. **b.** Heatmap and horizontal clustering of descriptors of Golgi morphology. U2OS cells were pre-treated with JNK and/or p38 inhibitors (JNKi, p38i, 0.5 h) and treated with anisomycin (Ani, 1 h) as indicated. Cells were fixed, immunostained with antibodies against *cis* Golgi marker GRASP65 and/or *trans* Golgi markers GRASP55 and TGN46 and images were acquired by high content microscopy. Images were processed and analyzed with CellProfiler software for calculation of the indicated parameters, and are presented as log2-transformed mean fold changes compared to the control. n>100 cells. **c.** Box plots of selected non-transformed parameters from (b). Dark grey represents conditions with activated p38 and JNK, light grey represents inhibitor-treated samples. Boxes show 25, 50 and 75 percentiles and whiskers show 10 and 90 percentiles. **d.** To dismantle the Golgi apparatus, U2OS cells were treated with Brefeldin-A (BFA – 1 h). This was done in the presence of ribosome inhibitors anisomycin or puromycin as indicated (after 0.5 h). BFA was washed out and cells were allowed to recover in the presence of ribosome inhibitors (2 h). Cells were fixed, immunostained with GRASP65 antibodies and counterstained with DAPI. Scale bars, 20 μm.
